# Vessel Wall-Derived Mesenchymal Stromal Cells Share Similar Differentiation Potential and Immunomodulatory Properties with Bone Marrow-Derived Stromal Cells

**DOI:** 10.1155/2020/8847038

**Published:** 2020-10-21

**Authors:** Zoltán Veréb, Anett Mázló, Attila Szabó, Szilárd Póliska, Attila Kiss, Krisztina Litauszky, Gábor Koncz, Zoltán Boda, Éva Rajnavölgyi, Attila Bácsi

**Affiliations:** ^1^Regenerative Medicine and Cellular Pharmacology Research Laboratory, Department of Dermatology and Allergology, University of Szeged, Szeged, Hungary; ^2^Research Institute of Translational Biomedicine, Department of Dermatology and Allergology, University of Szeged, Szeged, Hungary; ^3^Department of Immunology, Faculty of Medicine, University of Debrecen, Debrecen, Hungary; ^4^Doctoral School of Molecular Cellular and Immune Biology, University of Debrecen, Debrecen, Hungary; ^5^NORMENT, Centre of Excellence (CoE), Institute of Clinical Medicine, University of Oslo, and Division of Mental Health and Addiction, Oslo University Hospital, Oslo, Norway; ^6^Genomic Medicine and Bioinformatics Core Facility, Department of Biochemistry and Molecular Biology, Faculty of Medicine, University of Debrecen, Debrecen, Hungary; ^7^2nd Department of Internal Medicine, Faculty of Medicine, University of Debrecen, Debrecen, Hungary; ^8^Department of Vascular Surgery, Faculty of Medicine, University of Debrecen, Debrecen, Hungary

## Abstract

**Purpose:**

This study is aimed at investigating the phenotype, differentiation potential, immunomodulatory properties, and responsiveness of saphenous vein vessel wall-derived mesenchymal stromal cells (SV-MSCs) to various TLR ligands and proinflammatory cytokines, as well as comparing their features to those of their bone marrow-derived counterparts (BM-MSCs).

**Methods:**

SV-MSCs were isolated by enzymatic digestion of the saphenous vein vessel wall. Phenotype analysis was carried out by flow cytometry and microscopy, whereas adipogenic, chondrogenic, and osteogenic differentiation potentials were tested in *in vitro* assays. For comparative analysis, the expression of different stemness, proliferation, and differentiation-related genes was determined by Affymetrix gene array. To compare the immunomodulatory properties of SV-MSCs and BM-MSCs, mixed lymphocyte reaction was applied. To investigate their responses to various activating stimuli, MSCs were treated with TLR ligands (LPS, PolyI:C) or proinflammatory cytokines (TNF*α*, IL-1*β*, IFN*γ*), and the expression of various early innate immune response-related genes was assessed by qPCR, while secretion of selected cytokines and chemokines was measured by ELISA.

**Results:**

The isolated SV-MSCs were able to differentiate into bone, fat, and cartilage cells/direction *in vitro*. SV-MSCs expressed the most important MSC markers (CD29, CD44, CD73, CD90, and CD105) and shared almost identical phenotypic characteristics with BM-MSCs. Their gene expression pattern and activation pathways were close to those of BM-MSCs. SV-MSCs showed better immunosuppressive activity inhibiting phytohemagglutinin-induced T lymphocyte proliferation *in vitro* than BM-MSCs. Cellular responses to treatments mimicking inflammatory conditions were comparable in the bone marrow- and saphenous vein-derived MSCs. Namely, similar to BM-MSCs, SV-MSCs secreted increased amount of IL-6 and IL-8 after 12- or 24-hour treatment with LPS, PolyI:C, TNF*α*, or IL-1*β*, compared to untreated controls. Interestingly, a different CXCL-10/IP-10 secretion pattern could be observed under inflammatory conditions in the two types of MSCs.

**Conclusion:**

Based on our results, cells isolated from saphenous vein vessel wall fulfilled the ISCT's (International Society for Cellular Therapy) criteria for multipotent mesenchymal stromal cells, and no significant differences in the phenotype, gene expression pattern, and responsiveness to inflammatory stimuli could be observed between BM-MSCs and SV-MSCs, while the latter cells have more potent immunosuppressive activity *in vitro*. Further functional assays have to be performed to reveal whether SV-MSCs could be useful for certain regenerative therapeutic applications or tissue engineering purposes.

## 1. Introduction

Regeneration of blood vessels is essential for the homeostasis of vasculature as well as in the restoration of various forms of tissue injury. However, during inflammation or trauma, the endothelial layer of the vessels has limited regeneration potential. Furthermore, in many cases, the endothelium itself is responsible for maintaining the inflammation, which could lead to vessel malfunctions and tissue damage [1]. The remodeling of the vasculature is an intricately controlled collaboration among stem/progenitor cells, immune cells, and the residual cells of the vessel wall as well [2, 3]. This process is balanced by proangiogenic and antiangiogenic factors secreted by the cells mentioned above, and the vessels could be regenerated by circulating stem cells, stromal cells, endothelial progenitor cells, and vessel wall- or endothelium-related progenitor cells in the subendothelial tissue [2–4]. Various cell types and rare cell populations have the properties to differentiate to endothelial cells or to support the vasculogenic processes [5]; however, resident vascular stem/progenitor and stromal cells are thought to be a dominant subclass of the vascular wall cell population involved in vascular homeostasis, repair, and pathological processes [3, 6–13]. Mesenchymal stromal cells (MSCs) were first isolated from the bone marrow [14, 15], but over the last decades, cells with multilineage differentiation potential were also identified in many other organs and tissues, especially within the perivascular area of large vessels [16–18]. MSCs play a key role in the maintenance of tissue integrity and homeostasis due to their differentiation potential into another cell types and their immunomodulatory capacity as well. However, responses of MSCs to microbial stimuli, such as TLR ligands, or to proinflammatory cytokines are controversial topics, of which the details are yet to be elucidated.

In this study, we made an extended comparison of saphenous vein vessel wall-derived mesenchymal stem cells (SV-MSCs) and bone marrow-derived mesenchymal stem cells (BM-MSCs) regarding their phenotype, differentiation potential, immunomodulatory properties, and responsiveness to various activating stimuli.

## 2. Materials and Methods

### 2.1. Bone Marrow, Saphenous Vein, and Umbilical Cord Samples

Collections of bone marrow, umbilical cord, and saphenous vein samples complied with the directive of the Helsinki Declaration were approved by the institutional ethical review board (Medical Research Council) of the Medical and Health Science Center of the University of Debrecen (Ethical protocol numbers: UD MHSC REC/IEC No. 2754-2008, OSTRAT/1210-1/2008/OSTR). Tissue samples were collected corresponding to the EU Member States' Directive 2004/23/EC on tissue isolation [19].

For the isolation of BM-MSCs, approximately 10 ml of bone marrow aspirate was observed from the donors, which were diluted with saline in 1 : 3 ratio. The mononuclear cells were recovered by Ficoll Histopaque (Amersham Biosciences, Uppsala, Sweden) density gradient centrifugation. The number of live cells was determined by Trypan blue exclusion assay. Bone marrow nucleated cells were plated in 25 cm^2^ flasks at a density of 2 × 10^5^ living cells/cm^2^ and cultured in DMEM-LG medium (DMEM with 1 g/L glucose, Gibco/Invitrogen, London, UK), supplemented with 10% FSC and 1% Antibiotic-Antimycotic Solution (PAA Laboratories GmbH, Pasching, Austria). After 3-4 days, the nonadherent cells were removed, and the cultures were reefed with fresh medium. Thereafter, the cultures were fed every 3–4 days. When cells reached confluence, they were passaged (P1) after 0.025% trypsin-EDTA (both Sigma-Aldrich, Budapest, Hungary) application and replated into new 25 cm^2^ flasks. For positive BM-MSC control, MSCs from bone marrow were purchased from PromoCell (Heidelberg, Germany) and cultured under the same conditions. At passage P5, phenotypic analyses by flow cytometry, *in vitro* differentiation assays, and mycoplasma tests (Lonza, Basel, Switzerland) were performed. Cells positive for mycoplasma were excluded from the experiments.

Saphenous vein samples were collected from saphenectomies. The samples were collected and transported in ice cold PBS and processed within 4 hours. The vein was cleaned of adipose or connective tissue and then cut into small pieces. The segments were washed in PBS and then enzymatically digested by 0.2 mg/ml collagenase type XI (Sigma-Aldrich) dissolved in DMEM-LG medium for 60 minutes at 37°C. Cells were centrifuged at 1000 rpm for 20 minutes and washed by DMEM-LG medium. After two washing steps, cells were plated and cultured as described for BM-MSC.

The isolation and *in vitro* culture of human umbilical vein endothelial cells (HUVECs) were described elsewhere [20]. Briefly, HUVEC was removed from the umbilical cord with 1% collagenase type XI (Sigma-Aldrich) digestion and cultured in M199 medium (Sigma-Aldrich) supplemented with 20% FCS (Gibco, London, UK), 1% Antibiotic-Antimycotic Solution (PAA), and 1% L-glutamine (Gibco), in CO_2_ incubator at 37°C. After 4-5 days of culturing, when cells reached confluence, they were trypsinized and inoculated into new culture dishes. After 3 passages, the cell monolayers, which reached up to 70-80% confluence, were used for the experiments.

### 2.2. Flow Cytometry and Immunochemistry

A multiparameter analysis of the surface antigen expression of different MSCs, an HUVECs was performed by three-color flow cytometry using different fluorochrome-conjugated antibodies: CD34, CD44, CD45, CD49f, CD73, CD106, CD144, and CD147 (all from BD Biosciences, San Jose, CA, USA); CD49a (Biolegend, San Diego, CA, USA), CD14, CD29, CD31, CD36, CD47 CD49b, CD54, CD56, CD69, CD90, CD104, CD105, CD117, CD146, CD166, CXCR4, HLA-DR, PDGFRb, and VEGFR2 (all from R&D Systems, Minneapolis, MN, USA); and CD133 (Miltenyi Biotech, Gladbach, Germany). After harvesting the cells with 0.025% trypsin-EDTA, cells were washed with normal medium and then twice with FACS buffer. Cells were incubated with antibodies according the manufacturers' protocol on ice for 30 min then washed again with FACS buffer and fixed in 1% paraformaldehyde (PFA)/PBS and analyzed within 1 day. Samples were measured by a FACSCalibur flow cytometer (BD Biosciences Immunocytometry Systems, Franklin Lakes, NJ, USA), and data were analyzed using the FlowJo software (TreeStar, Ashland, OR, USA). Results were expressed as means of positive cells (%) ± SEM. For immunohistochemistry studies, cell cultures were fixed in 4% PFA; then, samples were labeled after washing the cells three times in PBS with primary antibodies against iNOS (Calbiochem/Merck, Merck Millipore, Darmstadt, Germany), von Willebrand factor (R&D Systems), and vimentin (Abcam, Cambridge, UK). Cell stainings were visualized with NorthernLights fluorochrome-conjugated secondary antibodies (R&D Systems). Actin filaments were stained with phalloidin-TRITC (Sigma-Aldrich). Nuclei were labeled with Hoechst 33342 (Invitrogen, Carlsbad, CA, USA) samples mounted with mounting medium-containing Mowiol (Merck) and glycerol in PBS and examined under an Olympus IX81 microscope equipped with a Hamamatsu Orca2 camera.

### 2.3. In Vitro Differentiation Assays

Adipogenic, chondrogenic, and osteogenic differentiations of MSC were performed by using Gibco's StemPro® Adipogenesis, Osteogenesis, and Chondrogenesis Differentiation Kits (Gibco). All differentiations were evaluated as per the manufacture's guide.

### 2.4. RNA Isolation, cDNA Synthesis, QPCR, and Microarray Data Analysis

Total RNA was isolated by TRIzol reagent (Invitrogen). 1.5-2 *μ*g of total RNA was reverse transcribed using SuperScript II RNase H reverse transcriptase (Invitrogen) and Oligo(dT)15 primers (Promega, Madison, WI, USA). Gene-specific TaqMan assays (Applied Biosystems) were used to perform QPCR in a final volume of 25 *μ*l in triplicates using AmpliTaq DNA polymerase and ABI Prism 7900HT real-time PCR instrument (Applied Biosystems). Amplification of 36B4 and/or cyclophylin was used as normalizing controls. Cycle threshold values (Ct) were determined using the SDS 2.1 software (Applied Biosystems). Constant threshold values were set for each gene throughout the study. The sequences of the primers and probes are available upon request.

To compare the gene expression profiles of different MSCs, Affymetrix GeneChip Human Gene 1.0 ST Arrays (Affymetrix, Santa Clara, CA, USA) were used as described previously [21]. Based on the literature, stem/stromal cell-related genes were selected, and statistical analysis was performed (Oneway ANOVA with Tukey post hoc test and Benjamini-Hochberg FDR) to calculate *p* value and fold change. To identify the relationships between the selected genes, the Ingenuity Pathway Analysis (IPA, Ingenuity Systems, Redwood City, CA, USA) was used. Excel datasheets containing gene IDs with the assigned gene expression values were uploaded into the program. The Ingenuity Pathways Knowledge Base (IPKB) provided all known functions and interactions which were published in the literature. For the representation of the relationships between the genes, the “Pathway Designer” tool of the IPA software was used.

### 2.5. Mixed Lymphocyte Reaction and Mitogen-Induced Cell Proliferation

Peripheral blood mononuclear cells (PBMCs) were isolated by a Ficoll gradient centrifugation (Amersham Biosciences). Prior to the test, 10^4^ and 10^5^ MSCs were placed in the cell culture plates, and nonadherent cells were removed by a gentle wash step. PBMCs required for the MLR test (1 × 10^6^) were added 24 hours later. Mitogen-activated T lymphocyte proliferation was induced by addition of concanavalin A (ConA) or phytohemagglutinin (PHA, both from Sigma-Aldrich) at a final concentration of 10 *μ*g/ml and 1 *μ*g/ml, respectively, to the MSC-PBMC cocultures. On day three, proliferation was detected by a BrDU colorimetric assay directly in the cell culture plate according to the manufacturer's instructions (Roche, Budapest, Hungary). In control experiments, MSCs and PBMCs were cultured together or separately with and without mitogenic activation. To compare the immunosuppressive capacity of SV-MSCs and BM-MSCs, the proliferation of mitogen-activated PBMCs (OD values, BrdU incorporation) was taken as value 1, and changes in BrdU incorporation caused by MSCs were compared.

### 2.6. In Vitro Activation of MSC

To investigate the role of TLR ligands and proinflammatory cytokines in MSCs, cells were plated to 24-well plates at 5 × 10^4^ cell density and then incubated with 100 ng/ml LPS (Sigma-Aldrich), 25 *μ*g/ml PolyI:C (InvivoGen, San Diego, CA, USA), 100 ng/ml TNF*α*, 10 ng/ml IFN*γ*, or 10 ng/ml IL-1*β* (all from Preprotech, Rocky Hill, NJ, USA). After the incubation, the supernatant was harvested and kept on -20°C until measurement. For qPCR measurements, cells were plated to 25 cm^2^ flasks and treated as mentioned above.

### 2.7. Measurement of Cytokine Secretion

Concentrations of secreted IL-6 cytokine as well as IL-8 and CXCL-10/IP-10 chemokines were measured using OptEIA kits (BD Biosciences) according to the manufacturer's protocol. Cell culture media were used as blank samples.

### 2.8. Statistical Analysis

The Statistica 7.0 software (StatSoft Inc., Tulsa, OK, USA) was used for the statistical analyses. Normality of distribution of data was assessed by Kolmogorov-Smirnov and Lilliefors tests. Nonnormally distributed parameters were transformed logarithmically to correct their skewed distributions. The R software was used for hierarchical clustering. Each experiment was performed at least three times, and each sample was tested in triplicate. Data are expressed as mean ± SD or SEM. Statistically significant difference was determined with two-way ANOVA analysis when there were more than two groups; for analysis between two groups, paired Student *t*-test was used. Significance level was set to 0.95; *p* values less than 0.05 (∗*p* < 0.05, ∗∗*p* < 0.01, ∗∗∗*p* < 0.001) were considered significant.

## 3. Results

### 3.1. Morphology, Differentiation Potential, and Phenotype of SV-MSCs

**Figure 1:**
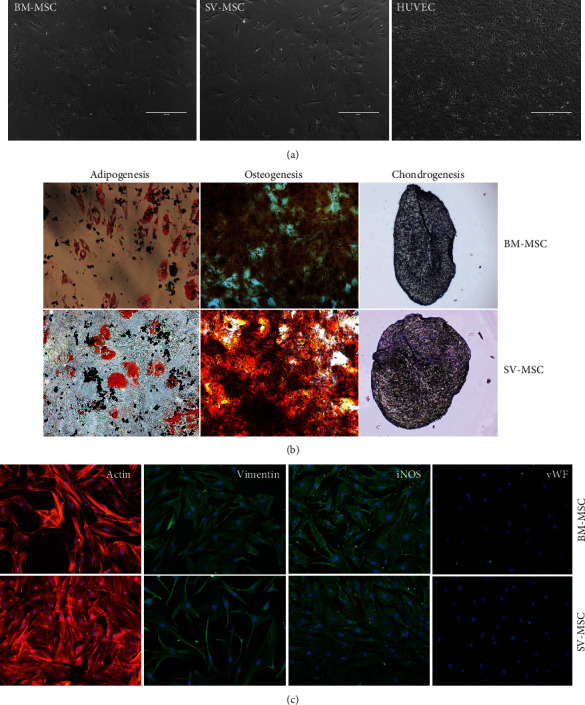
Comparison of morphology and multilineage differentiation potential of vessel wall- and bone marrow-derived MSCs. **(**a) After passage 5, the isolated MSC populations derived from bone marrow (BM) or SV and human umbilical cord vein (HUVEC) exhibited spindle-shaped morphology. (b) BM- and SV-derived MSCs exhibited the capability to differentiate into the three canonical differentiation pathways, such as fat, bone, and cartilage. (c) Cytoskeletal actin labeled by phalloidin-TRITC, vimentin, and iNOS by rabbit monoclonal antibody, visualized by anti-rabbit conjugated with NorthernLights493. Nuclei stained with Hoechst. Original magnification: ×200. Data is representative of four experiments.

MSCs isolated from saphenous vein showed similar morphology to bone marrow-derived MSCs (Figure 1(a)). The cultured cells never formed a cobblestone pattern, and their size was much larger than that of endothelial cells (HUVECs), which were used as vein endothelial cell controls in our experiments (Figure 1(a)). After 2-3 passages on adherent surface, the cells achieved uniform, fibroblast-like morphology, and these cells could be propagated at least for 15 passages without further morphological changes. An MSC type cell should fit the criteria defined by the Mesenchymal and Tissue Stem Cell Committee of the International Society for Cellular Therapy (ISCT) regarding plastic adherence, differentiation potential, and expression of cell surface markers [22]. In the following experiments, it was examined whether the SV-MSC cultures could be differentiated toward canonical mesodermal (adipogenic, osteogenic, chondrogenic) directions. Bone marrow-derived MSCs and SV-MSCs were differentiated *in vitro* using adipogenic, osteogenic, and chondrogenic induction media. Following three weeks of adipogenic differentiation induction, a large number of the SV-MSCs and BM-MSCs showed oil red positive staining, a characteristic for the adipocyte phenotype (Figure 1(b)). In parallel cell cultures, dense calcium deposits were detected after osteogenic differentiation (Figure 1(b)). In sections made from chondrogenic mass culture after 3 weeks of differentiation, metachromasy was observed upon toluidine-blue staining (Figure 1(b)). Both BM-MSC and SV-MSC cultures were positive for vimentin and iNOS; however, none of the cultures showed von Willebrand factor positivity, indicating the absence of endothelial cell contamination (Figure 1(c)).

For a detailed characterization, we compared the expression of cell surface markers on BM-MSCs and SV-MSCs by flow cytometry. As documented in Table 1, within the hematopoietic markers, no expression of CD34, CD45, CD69, CD133, and the CXCL12 receptor CXCR4 could be detected in the mesenchymal stromal cell cultures. A very small percentage of SV-MSCs was positive for CD117/c-kit (0.02 ± 0.02%), while none of the BM-MSCs expressed this marker. Neither BM-MSCs nor SV-MSCs expressed HLA-DR antigen-presenting molecule. Due to the possibility of endothelial contamination in SV-SMC cultures, we also investigated the expression of endothelial specific markers. CD31/PECAM, which makes up a large portion of endothelial cells, was absent both in the bone marrow- and saphenous vein-derived MSC cultures. The VEGFR2/KDR expression was very low in HUVEC cultures and was totally absent in MSC cultures. The expression of CD104/integrin *β*4 was more typical for the endothelial cells; however, it was also expressed on MSCs. The percentage of CD144/VE-Cadherin positive cells in SV-MSC cultures was in between those of endothelial cells and BM-MSCs (Table 1). Any cell that is described as mesenchymal stromal cell must fit the criteria defined by ISCT. All the expected markers such as CD73 (ecto-5′-nucleotidase**)**, CD90 (Thy-1), and CD105 (endoglin) could be detected both on BM-MSCs and SV-MSCs. Although CD73 and CD105 were also expressed by endothelial cells, the ratio of CD90 expressing cells was low in the HUVEC cultures (2.86 ± 1.55%), which distanced them from mesenchymal stromal cell identity. No statistically significant differences were found in the CD147 (neurothelin) and PDGFR*β* expression among the three cell types. None of the ISCT defined markers is exclusively MSC specific; therefore, we further investigated the expression of integrins and other cell adhesion molecules (CAMs), which determine the attachment and the fate of the cells within the tissues. Only the percentage of the melanoma cell adhesion molecule (CD146/MCAM) positive cells was found to be significantly different in bone marrow- (77.54 ± 5.14%) and saphenous vein-derived MSC cultures (7.09 ± 6.56%). Besides CD146, the expression of CD54/intercellular adhesion molecule 1 (ICAM-1), CD166/activated leukocyte cell adhesion molecule (ALCAM), CD56/neural cell adhesion molecule (NCAM), and CD44/homing-associated cell adhesion molecule (H-CAM) could be detected in the cell cultures; however, no significant differences were found in their expression. The expression of CD29/integrin (Itg) *β*1 and CD49a/Itg *α*1 was similar in BM-MSC, SV-MSC, and HUVEC cultures. In contrast, CD49b/Itg *α*2 was expressed at a lower level on the surface of vessel wall-derived MSCs, and the difference was found to be significant compared to HUVECs (*p* = 0.0186), but it was not significant compared to BM-MSCs. The CD49f/Itg *α*6 is mostly expressed by smooth muscle stromal cells, fibroblasts, and epithelial cells. MSCs isolated from either bone marrow or saphenous vein vessel wall did not show CD49f positivity (Table 1).

**Figure 2:**
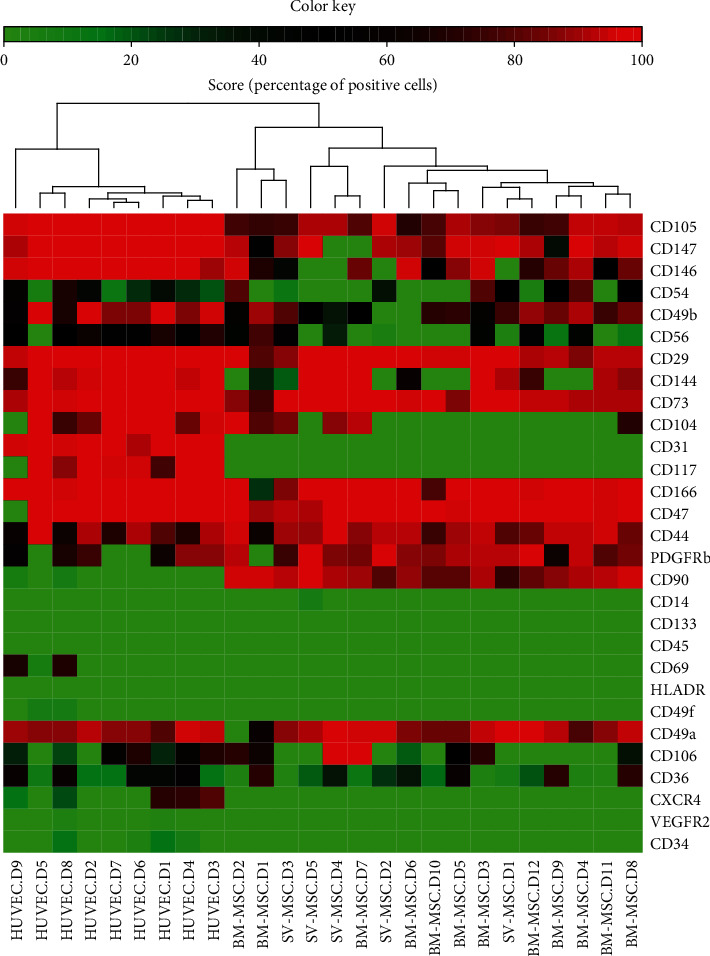
Hierarchical clustering of surface markers expressed by BM-MSCs and SV-MSCs. Robust hierarchical clustering of cell surface molecules' expression divided the BM-MSCs and SV-MSCs from the endothelial cells isolated from the umbilical cord tissue (HUVEC). SV-MSCs were more closely related to BM-MSC than to endothelial cells (a). (Color key represents percentage of positive cells in the in vitro cell cultures, *N*_HUVEC_ = 9, *N*_SV−MSC_ = 5, and *N*_BM−MSC_ = 12.)

Using a cluster analysis on the expression of the above surface markers in the three cell types, we found a clear division of the endothelial cells from the mesenchymal stromal cells (Figure 2). Results on SV-MSCs from different donors integrated well into the BM-MSC cluster despite interdonor variability. These observations indicate that our isolation technique with the applied phenotype analysis is suitable to detect mesenchymal stromal cells isolated from vessel wall (Figure 2).

### 3.2. Gene Expression Analysis

**Figure 3:**
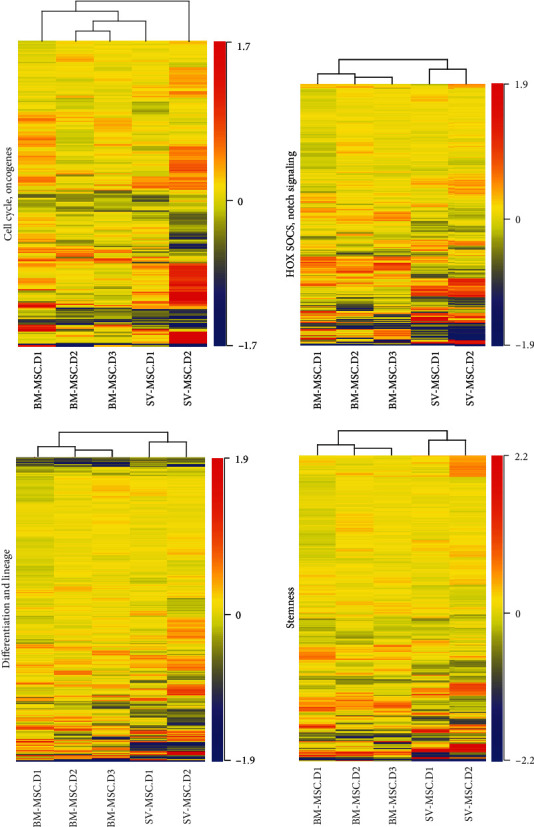
Heat maps of the differentially expressed genes in BM-MSCs and SV-MSCs. Genes related to stemness, HOX, Notch and SOX signaling, differentiation and lineage, cell cycle, and oncogenes were selected. The functional cluster analysis of the different expression levels of selected genes shows the difference between the cell types suggesting different tissue origin. (Color key represents relative gene expression levels.)

Next, the gene expression profiles of BM-MSCs and SV-MSCs were compared using microarray analyses. Genes related to differentiation and lineage (489 genes), stemness (422 genes), HOX (homeobox), SOCS (suppressor of cytokine signaling) and Notch signaling (380 genes), and cell cycle, oncogenes (242 genes) were collected into functional groups and analyzed. The hierarchical clustering clearly divided the cells with bone marrow and vessel wall origin in the case of differentiation and lineage, stemness and HOX, SOCS, and Notch signaling custom groups (Figure 3). The gene expression profile of SV-MSCs in the cell cycle and oncogenes custom group was not significantly different from that of their BM-MSC counterpart (Figure 3); however, several genes related to this biological function group were differentially expressed in BM-MSCs and SV-MSCs (Table 2). In SV-MSCs, expression level of *S100A4* (S100 calcium binding protein A4) was significantly higher (2.8-fold change), whereas that of *SMAD3* (SMAD family member 3) and *CDK6* (cyclin-dependent kinase 6) was significantly lower (-2.6- and -2.2-fold change, respectively) than in BM-MSCs (Table 2). Significantly upregulated (≥2-fold) genes related to differentiation in SV-MSCs were found to be *PODXL* (podocalyxin-like), *CTSK* (cathepsin K), and *CSF1* (colony stimulating factor 1/macrophage), while *VCAM1* (vascular cell adhesion molecule 1), *ACAN* (aggrecan), *EGR2* (early growth response 2), *TGFB2* (transforming growth factor beta 2), *IGF2* (insulin-like growth factor 2), *BMP2* (bone morphogenetic protein 2), *BDNF* (brain-derived neurotrophic factor), *JAG1* (jagged 1), *INHBA* (inhibin, beta A), *ITGA3* (integrin, alpha 3), *SMAD3*, *HES1* (hairy and enhancer of split 1), *EFNB2* (ephrin-B2), *PTN* (pleiotrophin), and *PDGFA* (platelet-derived growth factor alpha) genes were significantly downregulated (≤ -2-fold). An SV-MSC-specific pattern of stemness could be characterized with high expression of *FGF9* (fibroblast growth factor 9 or glia-activating factor), *ZFPM2* (zinc finger protein, multitype 2), *MME* (membrane metallo-endopeptidase), and *FZD4* (frizzled homolog 4) genes, together with low expression of *LIF* (leukemia inhibitory factor or cholinergic differentiation factor), *MGC20647* (hypothetical protein MGC20647), *CXCL12* (chemokine C-X-C motif ligand 12 or stromal cell-derived factor 1), *MCAM* (melanoma cell adhesion molecule), *ACAN*, *LTBP1* (latent transforming growth factor beta binding protein 1), *BMP2*, *SMAD3*, *ALCAM*, *ITGAV* (integrin, alpha V, or vitronectin receptor), *GDF6* (growth differentiation factor 6), and *FGF7* (fibroblast growth factor 7) genes (Table 2). In the HOX, SOCS, and Notch signaling, superfamily *FGF9*, *IL-33* (interleukin-33), and *HOXA11* (homeobox A11) genes were determined as significantly upregulated (≥2-fold) ones in SV-MSCs (Table 2). Focusing on the expression of MSC-related genes, our microarray data were validated by a qPCR-based gene array as well (Supplementary Figure 1). During characterization of MSCs, the study of senescence is particularly important. Our gene expression analysis revealed that only 18 out of 160 genes known to be important in the development of senescence are expressed differently in BM- and SV-MSCs (Supplementary Figure 2 and Supplementary Table 1). *CHEK2*, *GAA*, *TP53*, *CDKN1A*, *SQSTM1*, *CTSB*, *GLB1*, *ATP6V1G2*, and *ETS2* genes were upregulated, while *MCL1*, *CDK6*, *BAT1*, *TGFB1*, *FN1*, *PLAU*, *GALNT5*, *IGF1R*, and *IGFBP7* genes were downregulated in SV-MSCs compared to BM-MSCs. Most of the upregulated genes are responsible for maintaining replicative capacity and inhibiting cellular senescence. Taken together, our data suggest that BM-MSCs and SV-MSCs display very similar pattern of the gene expression.

### 3.3. Immunomodulatory Properties of BM-MSCs and SV-MSCs

**Figure 4:**
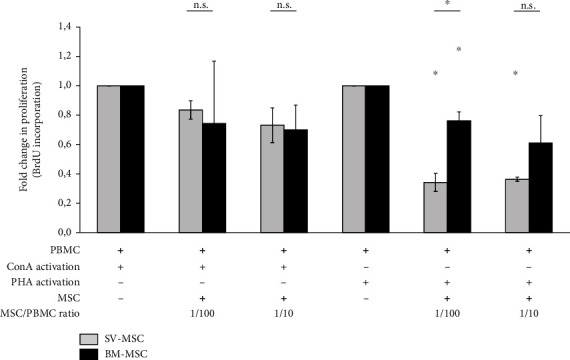
Immunomodulatory effect of SV- and BM-MSCs *in vitro*. Fold change in PBMCs' proliferation showed that bone marrow- and vessel wall-derived MSCs were capable to inhibit the proliferation of lymphocytes activated by either PHA or ConA *in vitro*; however, statistically significant reductions in BrDU incorporation were observed only in the case of PHA. (Data shown are mean ± SEM, *N* = 3; *p* < 0.05^∗^.)

The immunosuppressive properties of MSCs have been extensively studied over the past years, for their promising clinical application potential. In the present study, mitogenic mixed lymphocyte reaction (MLR) was used to compare the immunosuppressive properties of BM-MSCs and SV-MSCs. Human PBMCs from healthy donors were applied as responder cells and ConA or PHA as mitogenic activators. As expected, PBMCs proliferated in response to ConA or PHA treatment (data not shown). The addition of either BM-MSCs or SC-MSCs to PBMCs stimulated with ConA resulted in a moderate, statistically insignificant reduction of BrDU incorporation (Figure 4). In contrast, statistically significant suppressions of lymphocyte proliferation by both BM-MSCs and SV-MSCs could be detected in PBMCs cultures activated with PHA (Figure 4). At both cell ratios (MSC/PBMC 1 : 100 and 1 : 10), the suppression of PBMC proliferation by SV-MSCs was more prominent; however, a significant difference between SV-MSCs and BM-MSCs in their suppressive effects was observed only at a ratio of 1 : 100 (Figure 4).

### 3.4. Activation of BM-MSCs and SV-MSCs

**Figure 5:**
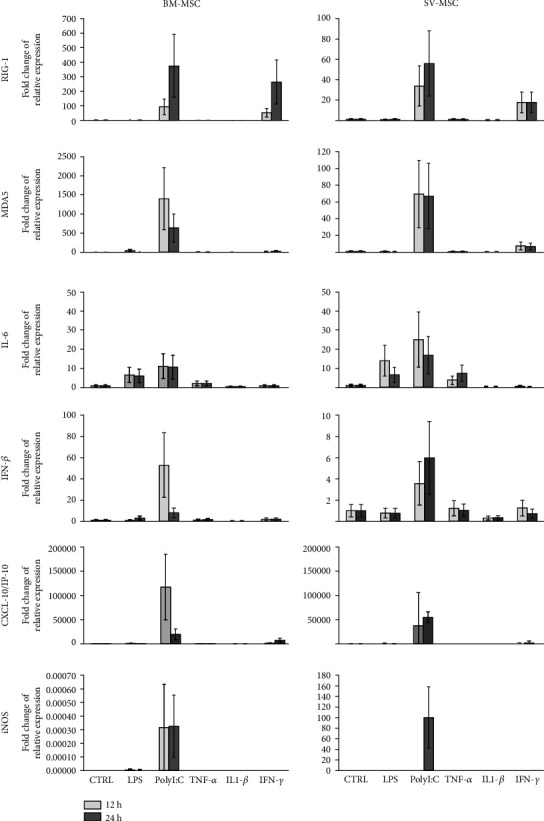
Relative expression levels of selected genes in MSCs derived from bone marrow and saphenous vein in resting and stimulated cells. Mesenchymal stromal cells were treated with 100 ng/ml LPS, 25 *μ*g/ml PolyI:C, 100 ng/ml TNF*α*, 10 ng/ml IL-1*β*, or 10 ng/ml IFN*γ* for 12 and 24 hours as described in Methods. Relative levels of mRNA were measured in triplicates by qPCR, and fold changes in the gene expression were calculated from the ratio of expression levels in treated and untreated cells as mean ± SEM (*N* = 3 in both cell types).

Although the immunosuppressive function of MSCs is well described, much fewer details are available about their response to proinflammatory cytokine exposure or TLR ligand activation, especially in case of vessel wall-derived MSCs. Therefore, in the next series of our experiments, BM-MSCs and SV-MSCs were treated with LPS, PolyI:C, TNF*α*, IL-1*β*, or IFN*γ* for 12 and 24 hours, and the expression of various early innate immune response-related genes was investigated. As shown in Figure 5, the mRNA expression level of a dsRNA sensor *RIG-I* (retinoic acid-inducible gene I) was increased after 12 and 24 hours upon PolyI:C and IFN*γ* treatment both in BM-MSCs and SV-MSCs. Activation with PolyI:C also induced a marked rise in the expression of *MDA5*, another dsRNA sensor of the RIG-I-like receptor family [23]. Induction of both *RIG-I* and *MDA5* gene expression was more robust in BM-MSCs. The expression of *IL-6* gene was increased upon LPS, PolyI:C, and TNF*α* treatments at both time points in both MSC types. The *IFNβ* expression was markedly upregulated in the case of PolyI:C activation following 12- and 24-hour treatments in both type of MSCs. A robust increase in the gene expression level of CXCL-10/IP-10 (interferon gamma-induced protein 10) was observed when MSCs were treated with PolyI:C at both time points (Figure 5). Inducible nitrogen-oxide synthase (iNOS) is a key element of MSC-mediated immunosuppression [24]. The expression of *iNOS* was notably induced in SV-MSCs after a 24-hour PolyI:C treatment, whereas its expression level in BM-MSCs remained almost unchanged under the same conditions. Overall, there were no significant differences between SV-MSCs and BM-MSCs in the expression pattern of genes associated with TRL ligand- and proinflammatory cytokine-triggered activation (Supplementary Figure 3).

**Figure 6:**
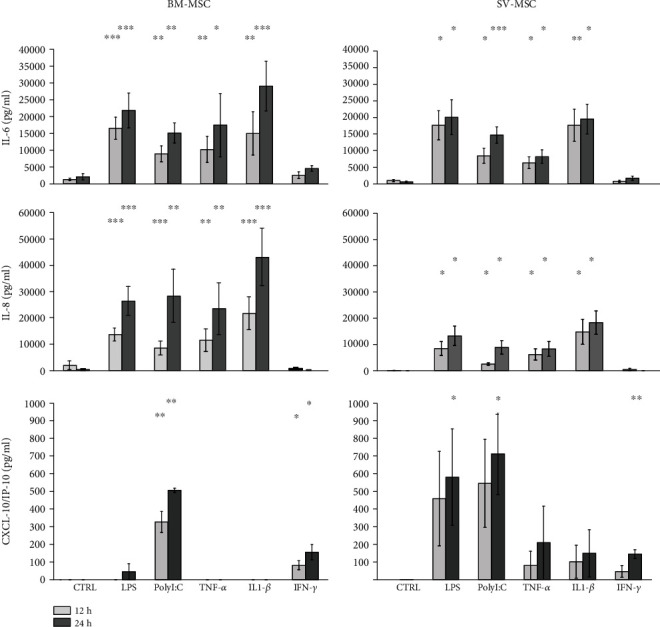
Cytokine secretion by activated MSCs derived from bone marrow and saphenous vein. IL-6, IL-8, and IP-10 cytokine production of TRL ligands (LPS, PolyI:C), as well as proinflammatory cytokine- (TNF*α*, IFN*γ*, IL-1*β*) stimulated MSCs. In vitro cultured cells were treated in 12 h and 24 h intervals. (Data shown are mean ± SD; *p* < 0.05^∗^, *p* < 0.01^∗∗^, *p* < 0.001^∗∗∗^; *N* = 6 for the BM-MSCs and *N* = 3 for the SV-MSCs, respectively.)

To validate our findings at protein level, secreted cytokine and chemokine concentrations were also determined in MSC cultures upon activation of the cells with TLR ligands or proinflammatory cytokines (Figure 6). IL-6 was constantly secreted by both types of MSCs under normal conditions. Secretion patterns of IL-6 cytokine and IL-8 chemokine were similar in both MSC cultures. Exposure to LPS, PolyI:C, TNF*α*, or IL-1*β* for 12 and 24 hours triggered a significant increase in the concentrations of both above mentioned secreted mediators, whereas treatments with IFN*γ* did not modify their production by BM-MSCs and SV-MSCs. More intense IL-6 and IL-8 production was observed in BM-MSC than SV-MSC culture (Figure 6). Both type of MSCs secreted CXCL-10/IP-10 chemokine upon TLR- and cytokine receptor ligation. In contrast to IL-6 and IL-8 levels, SV-MSCs produced more CXCL-10/IP-10 in response to activation than BM-MSCs. Based on our measurements, in BM-MSCs cultures, PolyI:C and IL-1*β* stimuli were the most potent inducers of CXCL-10/IP-10 production, while SV-MSCs released this chemokine in increased concentrations as a result of any applied activations; however, the changes were statistically significant only when the cells were exposed to the TLR ligands, LPS, or PolyI:C (Figure 6).

## 4. Discussion

Mesenchymal stromal cells reside in various tissues of mesodermal origin. They are multipotent cells, which are able to differentiate into various types of specialized cells including osteoblasts, chondrocytes, and adipocytes [25]. This ability endows MSCs with a broad regenerative potential in adult tissues [26]. However, MSCs do not only contribute to tissue repair processes but also have strong immunomodulatory properties and may inhibit inflammation by modulation of local environment [27].

In this study, we separated MSCs from saphenous vein vessel wall and compared their morphology, phenotype, and functions to those of bone marrow-derived MSCs to reveal the differences and similarities, which could be associated with their regulatory role in angiogenesis or their immunomodulatory properties under physiologic and pathologic conditions. Pericytes and MSCs share morphology, expression of several cell surface molecules, and even differentiation potential *in vitro*; however, MSCs can be characterized by a combination of perivascular (CD146, PDGFR*β*) and MSC markers (CD29, CD44, CD73, CD90, CD105) as well as by the lack the expression of hemato-endothelial cell markers (CD31, CD34, CD45, CD144) [28]. According to our findings, the cells isolated from saphenous vein (SV-MSC) showed similar morphology to bone marrow-derived MSCs (BM-MSC). The plastic adherent MSCs have been shown to differentiate toward multiple mesodermal lineages including fat, bone, and cartilage cells. SV-MSC cultures could be differentiated toward canonical mesodermal; adipogenic, osteoblastic, and chondrogenic directions culturing the cells in the appropriate induction media. Similar to BM-MSCs, the SV-MSCs are also fit to the criteria defined by ISCT, which means that all expected markers could be detected. The populations of BM- and SV-MSCs were well identified; they differed from the myoblasts, smooth muscle cell precursors, or from the control HUVEC cells. The only significant difference identified was the higher expression of CD146 on the surface of BM-MSCs than on that of SV-MSCs. Previous findings in *in vitro* and animal models suggest that higher CD146 level on MSCs is associated with more plasticity, better ability for transendothelial migration but lower regenerative potential of the cells [29, 30]. Further studies examining the human relevance of these observations need to be performed.

Our results provide an evidence that the isolation technique invented by our group is suitable to collect a pure vessel wall-derived mesenchymal stromal cell population. To investigate differences and similarities between the gene expression profile of BM-MSCs and SV-MSCs, we examined the genes related to differentiation and lineage, stemness, HOX, SOCS, Notch signaling, cell cycle, and oncogenes. These data were collected into functional groups to reveal and compare the functional properties of the BM-MSCs and SV-MSCs. According to the hierarchical clustering in case of the genes related to the cell cycle and oncogenes custom group, we did not detect any significant difference between the BM- and SV-MSCs. In contrast, the expression profile clearly divided the cells isolated from bone marrow and saphenous vein into two groups in case of the differentiation and lineage, stemness and HOX, SOCS, and Notch signaling groups as well as senescence. Above described differences could be the consequences of the variant origin and localisation of MSCs [31], functions of BM-MSCs in the bone marrow to support the differentiation and survival of hematopoietic stem cells (HSC) while the SV-MSCs are responsible for the regeneration and wound healing, angiogenesis, and neovascularization [32]. The immunosuppressive activity of BM-MSCs is already published in details underlying their importance in the treatment or their possible application in case of a wide array of nonphysiologic conditions like autoimmune and inflammatory diseases or cancer. Based on our present data, we can state that SV-MSCs also have a potential to suppress the mitogenic activation of PBMCs. Moreover, in our experiments, SV-MSCs displayed better immunosuppressive activity inhibiting PHA-induced T lymphocyte proliferation *in vitro* than BM-MSCs.

When MSCs are exposed to degraded ECM products, they exhibited an increased migratory capability [33]. These changes prove the activation of MSCs in the presence of various stimuli such as injury, infection, or sterile inflammation resulted in the enhanced secretion of various cytokines, like basic-fibroblast growth factor (b-FGF), chemotactic and mitogenic molecules, or vascular endothelial growth factor (VEGF) modulating the angiogenesis [34]. Like many other cells, the MSCs also express extra- and intracellular pattern recognition receptors (PRRs). Immunomodulatory functions of MSCs can be influenced by either ligation of PRRs or via exposure to cytokines and other immunomodulatory factors [35]. Response of MSCs to different stimulatory factors determinates the differentiation and functions of neighbouring immune and not immune cells thus the immune responses themselves [36, 37]. Both BM- and SV-MSCs could be stimulated by PolyI:C leading to the increased expression of RIG-I, MDA5, IL-6, IFN*β*, CXCL-10/IP-10, and iNOS. However, we detected differences in the intensity of cellular responses following the PolyI:C treatments. BM-MSCs are able to react to PolyI:C to a greater extent by expressing higher levels of RIG-I, MDA-5, IFN*β*, and CXCL-10/IP-10 than SV-MSCs. In agreement with previous findings [38], both BM-MSCs and SV-MSCs could be activated with LPS despite the fact that MSCs express CD14, which plays a vital role in TLR4 signaling pathway, at a very low level. LPS treatment may induce slight expression of cytokines and chemokines in MSCs without activation of AKT, NF-*κ*B, and P38 [38]. TLR and cytokine receptor ligation resulting in upregulated secretion of IL-6, IL-8, and CXCL-10/IP-10, although a treatment with IFN*γ* had no effect on their cytokine and chemokine production. Priming of MSCs by PRR ligands to alter their immunomodulatory activity is known to be essential to use these cells in the treatment of various diseases [39]; however, it seems that MSCs isolated from bone marrow or saphenous vein respond to priming stimuli in slightly different ways.

Under physiologic and pathologic conditions, MSCs express a wide array of surface markers and produce various factors by which they can communicate with different cell types including immune cells. The immunomodulatory capacity of MSCs may result in the inhibited proliferation of lymphocytes and suppressed function of activated inflammatory cells. Furthermore, they are able to drive and determine the differentiation of myeloid-derived cells and the polarization of the T cell response [40]. Based on our results, SV-MSCs fulfill the ISCT criteria for multipotent mesenchymal stromal cells and share almost identical phenotypic and functional characteristics with BM-MSCs. Furthermore, SV-MSCs are easy to obtain and could be alternative sources of MSCs with tissue origin. Therefore, SV-MSCs can be considered as good candidate for further thorough investigations (e.g., assays testing proliferative capacity, sensitivity to apoptosis, and karyotype changes during replication) to reveal whether these cells could be useful for certain regenerative therapeutic and tissue engineering applications (e.g., 3D bioprinting).

## Figures and Tables

**Table 1:** Detailed phenotypic analysis of BM-MSCs and SV-MSCs.

		BM-MSC	SV-MSC	HUVEC
		Percentage of positive cells (%)		
Hematopoietic markers	CD14	0.22 ± 0.11	1.37 + 1.15	0 + 0
	CD34	0 ± 0	0 ± 0	4.62 + 2.05
	CD36	32.51 ± 8.18	18.12 ± 5.28	36.6 + 17.60
	CD45	0 ± 0	0 ± 0	0 + 0
	CD47	97.00 ± 0.86	96.65 ± 1.55	85.06 + 12.49
	CD69	0 ± 0	0 ± 0	27.24 + 10.93
	CD133	0 ± 0	0 ± 0	0 + 012.03
	CD117	0 ± 0	0.02 ± 0.02	81.57 + 11.26^∗∗∗^
	CXCR4	0 ± 0	0 ± 0	37.37 + 8.18^∗∗^
	HLA-DR	0 ± 0	0 ± 0	0.19 + 0.12

Endothelial markers	CD31	0 ± 0	0 ± 0	96.78 + 0.82^∗∗∗^
	CD144	45.33 ± 12.61	61.55 ± 18.18	93.91 + 2.45
	VEGFR2/KDR	0 ± 0	0 ± 0	0.75 + 0.41
	CD104/integrin *β*4	28.25 ± 12.20	34.42 ± 17.82	76.42 + 11.50

MSC/fibroblast markers	CD73	91.99 ± 1.92	97.90 ± 0.80	97.85 + 0.94
	CD90/Thy-1	89.05 ± 1.49	89.68 ± 3.63	2.86 + 1.55^∗∗∗^
	CD105/endoglin	82.64 ± 2.56	89.62 ± 2.54	97.94 + 0.52^∗∗∗^
	CD147/neurothelin	77.33 ± 8.87	81.11 ± 13.59	98.31 + 0.91
	PDGF R*β*	78.01 ± 8.28	90.77 ± 3.74	54.67 + 11.90

Cell adhesion molecules	CD29/integrin *β*1	92.96 ± 1.71	97.02 ± 1.87	98.77 + 0.64
	CD44/H-CAM	87.28 ± 2.87	88.66 ± 2.38	79.28 + 5.06
	CD49a	79.60 ± 7.77	94.25 ± 1.55	89.44 + 1.64
	CD49b	68.52 ± 7.95	48.44 ± 12.25	85.32 + 5.42^∗^
	CD49f	0 ± 0	0 ± 0	2.21 + 1.27
	CD54/ICAM	14.95 + 8.36	19.89 + 8.59	34.29 + 7.24
	CD56/NCAM	20.53 ± 8.41	19.18 ± 9.10	50.33 + 7.94
	CD146/MCAM	77.54 ± 5.14^∗∗∗^	7.09 ± 6.56	96.68 + 1.02^∗∗∗^
	CD166/ALCAM	89.57 ± 6.27	96.22 ± 2.13	98.54 + 0.45

Expression of surface markers related to different cell types was measured by flow cytometry. The percentage of positive cells in SV-MSC culture was compared to that of BM-MSCs as well as HUVECs, as vein endothelial control. (Data are presented as means ± SEM; *N* = 5 for SV-MSC, *N* = 12 for BM-MSC, *N* = 7 for HUVEC. *p* < 0.05^∗^, *p* < 0.01^∗∗^, *p* < 0.001^∗∗∗^ vs. SV-MSCs determined by Student *t*-test).

**Table 2:** Top up- and downregulated custom selected genes in SV-MSCs.

Symbol	Entrez gene name	Fold change	*p* value	Molecule type	Group
Fold change upregulated					
*S100A4*	S100 calcium binding protein A4	2.805862	0.0426795	Calcium binding protein	Cell cycle and oncogenes
*CDH1*	Cadherin 1, type 1, E-cadherin (epithelial)	1.61272	0.0221517	Cell adhesion molecule	
*BRCA2*	Breast cancer 2, early onset	1.36718	0.0158221	DNA repair	
*SMG6*	Smg-6 homolog, nonsense mediated mRNA decay factor (C, elegans)	1.1617644	0.0169296	Enzyme	

*FGF9*	Fibroblast growth factor 9 (glia-activating factor)	6.6313844	0.0116976	Growth and differentiation factor	HOX, SOCS, Notch signaling
*IL-33*	Interleukin-33	2.8804057	0.0380778	Cytokine	
*HOXA11*	Homeobox A11	2.00343	0.0164048	Transcription factor	
*BMP4*	Bone morphogenetic protein 4	1.8365396	0.0429056	Growth and differentiation factor	

*PODXL*	Podocalyxin-like	4.6124125	0.0230397	Cell differentiation	Differentiation and lineage
*CTSK*	Cathepsin K	2.5564253	0.0201834	Lysosomal cysteine protease	
*CSF1*	Colony stimulating factor 1 (macrophage)	2.176855	0.0155533	Cytokine	
*TGFB3*	Transforming growth factor, beta 3	1.8547666	0.0230397	Growth and differentiation factor	
*NRP1*	Neuropilin 1	1.7981821	0.0058021	Membrane-bound coreceptor	
*GDF10*	Growth differentiation factor 10	1.6743402	0.0155533	Growth and differentiation factor	

*FGF9*	Fibroblast growth factor 9 (glia-activating factor)	6.6313844	0.0143408	Growth and differentiation factor	Stemness
*ZFPM2*	Zinc finger protein, multitype 2	5.1527076	0.0195738	Transcription factor	
*MME*	Membrane metallo-endopeptidase	3.1862447	0.0245681	Enzyme	
*FZD4*	Frizzled homolog 4 (drosophila)	2.4862442	0.0143408	Receptor	
*ACVRL1*	Activin A receptor type II-like 1	1.8575617	0.0143408	Enzyme	

Fold change downregulated					
*SMAD3*	SMAD family member 3	-2.579574	0.0108465	Transcriptional modulator	Cell cycle and oncogenes
*CDK6*	Cyclin-dependent kinase 6	-2.243104	0.0237225	Enzyme	
*KRAS|LYRM5*	v-Ki-ras2 Kirsten rat sarcoma viral oncogene homolog|LYR motif containing 5	-1.901833	0.0461384	Proto-oncogene	
*TGFB1*	Transforming growth factor, beta 1	-1.731404	0.0158221	Growth and differentiation factor	
*RARA*	Retinoic acid receptor, alpha	-1.566421	0.0337291	Nuclear receptor	
*HUS1*	Checkpoint protein HUS1	-1.287702	0.0108465	Genotoxin-activated checkpoint complex	
*SUN1|C7orf20*	Sad1 and UNC84 domain containing 1|chromosome 7 open reading frame 20	-1.263087	0.0108465	Nuclear envelope protein	
*PURA*	Purine-rich element binding protein A	-1.253684	0.0108465	Multifunctional DNA- and RNA-binding protein	

*CDON*	Cdon homolog (mouse)	-1.5534242	0.0691849	Cell surface receptor	HOX, SOCS, Notch signaling
*HOXA2*	Homeobox A2	-1.6179696	0.0280393	Transcription factor	
*SNAI1*	Snail homolog 1 (drosophila)	-1.6272678	0.0442530	Transcription factor	
*PYGO1*	Pygopus homolog 1 (drosophila)	-1.6621072	0.0924799		
*NOTCH2*	Notch homolog 2 (drosophila)	-1.6733813	0.0177410	Transmembrane protein	
*MAML2*	Mastermind-like 2 (drosophila)	-1.6876673	0.0481715	Transcriptional coactivator	

*VCAM1*	Vascular cell adhesion molecule 1	-17.354261	0.0058021		Differentiation and lineage
*ACAN*	Aggrecan	-5.5746202	0.0192411		
*EGR2*	Early growth response 2	-4.387574	0.0191151		
*TGFB2*	Transforming growth factor, beta 2	-3.9135396	0.0058021		
*IGF2|INS-IGF2*	Insulin-like growth factor 2 (somatomedin A)|INS-IGF2 readthrough transcript	-3.4904327	0.0192411		
*BMP2*	Bone morphogenetic protein 2	-3.314717	0.0230696		
*BDNF*	Brain-derived neurotrophic factor	-3.2864723	0.0155533		
*JAG1*	Jagged 1 (Alagille syndrome)	-3.0462105	0.0058021		
*INHBA*	Inhibin, beta A	-2.8667028	0.0422998		
*ITGA3*	Integrin, alpha 3 (antigen CD49C, alpha 3 subunit of VLA-3 receptor)	-2.775381	0.0155533		
*SMAD3*	SMAD family member 3	-2.5795743	0.0078861		
*HES1*	Hairy and enhancer of split 1 (drosophila)	-2.220433	0.0192411		
*EFNB2*	Ephrin-B2	-2.1113176	0.0358564		
*PTN*	Pleiotrophin	-2.1063795	0.0155533		
*PDGFA LOC100132080*	Platelet-derived growth factor alpha polypeptide|hypothetical LOC100132080	-2.0340111	0.0155533		

*LIF|MGC20647*	Leukemia inhibitory factor (cholinergic differentiation factor)|hypothetical protein MGC20647	-9.517681	0.0154874		Stemness
*CXCL12*	Chemokine (C-X-C motif) ligand 12|chemokine (C-X-C motif) ligand 12 (stromal cell-derived factor 1)	-8.499458	0.0414186		
*MCAM*	Melanoma cell adhesion molecule	-5.909656	0.0080718		
*ACAN*	Aggrecan	-5.5746202	0.0193145		
*LTBP1*	Latent transforming growth factor beta binding protein 1	-4.3929467	0.0398936		
*BMP2*	Bone morphogenetic protein 2	-3.314717	0.0236414		
*SMAD3*	SMAD family member 3	-2.5795743	0.0087946		
*ALCAM*	Activated leukocyte cell adhesion molecule	-2.1179285	0.0080718		
*ITGAV*	Integrin, alpha V (vitronectin receptor, alpha polypeptide, antigen CD51)	-2.0977302	0.0143408		
*GDF6*	Growth differentiation factor 6	-2.054666	0.0427049		
*FGF7*	Fibroblast growth factor 7 (keratinocyte growth factor)	-2.0363815	0.049803		
*FGFR2*	Fibroblast growth factor receptor 2	-1.8906314	0.0324610		

Top up- and downregulated genes in SV-MSCs related to stemness, HOX, Notch and SOX signaling, differentiation and lineage, cell cycle, and oncogenes were selected by the significance.

## Data Availability

The gene array data used to support the findings of this study are available from the corresponding author upon request.

## Supplementary Materials

Supplementary 1 Figure S1: Heat map of differently expressed genes related to MSC and endothel cell biology.

Supplementary 2 Figure S2: Expression of genes associated with cellular senescence

Supplementary 3 Figure S3: Heat maps of differently expressed genes related to TRL ligand- and proinflammatory cytokine-triggered activation.

Supplementary 4 Supplementary Table 1: Significantly expressed genes associated with cellular senescence in SV-MSCs and BM-MSCs.

## References

[1] Higashi Y., Kihara Y., Noma K. (2012). Endothelial dysfunction and hypertension in aging. *Hypertension Research*.

[2] Wietecha M. S., Cerny W. L., DiPietro L. A. (2012). Mechanisms of vessel regression: toward an understanding of the resolution of angiogenesis. *New Perspectives in Regeneration*.

[3] Wanjare M., Kusuma S., Gerecht S. (2013). Perivascular cells in blood vessel regeneration. *Biotechnology Journal*.

[4] Korta K., Kupczyk P., Skóra J. (2013). Stem and progenitor cells in biostructure of blood vessel walls. *Postȩpy Higieny i Medycyny Doświadczalnej*.

[5] Crisan M., Deasy B., Gavina M. (2008). Purification and long-term culture of multipotent progenitor cells affiliated with the walls of human blood vessels: myoendothelial cells and pericytes. *Methods in Cell Biology*.

[6] Gómez-Gaviro M. V., Lovell-Badge R., Fernández-Avilés F., Lara-Pezzi E. (2012). The vascular stem cell niche. *Journal of Cardiovascular Translational Research*.

[7] Torsney E., Xu Q. (2011). Resident vascular progenitor cells. *Journal of Molecular and Cellular Cardiology*.

[8] Xu Q. (2007). Progenitor cells in vascular repair. *Current Opinion in Lipidology*.

[9] Watt S. M., Athanassopoulos A., Harris A. L., Tsaknakis G. (2010). Human endothelial stem/progenitor cells, angiogenic factors and vascular repair. *Journal of The Royal Society Interface*.

[10] Hagensen M. K., Vanhoutte P. M., Bentzon J. F. (2012). Arterial endothelial cells: still the craftsmen of regenerated endothelium. *Cardiovascular Research*.

[11] Curcio A., Torella D., Indolfi C. (2011). Mechanisms of smooth muscle cell proliferation and endothelial regeneration after vascular injury and stenting: approach to therapy. *Circulation Journal*.

[12] Crisan M., Corselli M., Chen C. W., Péault B. (2011). Multilineage stem cells in the adult: a perivascular legacy?. *Organogenesis*.

[13] Critser P. J., Yoder M. C. (2010). Endothelial colony-forming cell role in neoangiogenesis and tissue repair. *Current Opinion in Organ Transplantation*.

[14] Friedenstein A. J., Deriglasova U. F., Kulagina N. N. (1974). Precursors for fibroblasts in different populations of hematopoietic cells as detected by the in vitro colony assay method. *Experimental Hematology*.

[15] Friedenstein A. J., Chailakhyan R. K., Latsinik N. V., Panasyuk A. F., Keiliss-Borok I. V. (1974). Stromal cells responsible for transferring the microenvironment of the hemopoietic tissues. Cloning in vitro and retransplantation in vivo. *Transplantation*.

[16] Pasquinelli G., Pacilli A., Alviano F. (2010). Multidistrict human mesenchymal vascular cells: pluripotency and stemness characteristics. *Cytotherapy*.

[17] Yang S., Eto H., Kato H. (2013). Comparative characterization of stromal vascular cells derived from three types of vascular wall and adipose tissue. *Tissue Engineering. Part A*.

[18] Majesky M. W., Dong X. R., Hoglund V., Daum G., Mahoney, Jr W. M. (2012). The adventitia: a progenitor cell niche for the vessel wall. *Cells, Tissues, Organs*.

[19] European Union (2004). Directive 2004/23/EC of the European Parliament and of the Council of 31 March 2004 on setting standards of quality and safety for the donation, procurement, testing, processing, preservation, storage and distribution of human tissues and cells. *OJ L*.

[20] Palatka K., Serfozo Z., Vereb Z. (2006). Effect of IBD sera on expression of inducible and endothelial nitric oxide synthase in human umbilical vein endothelial cells. *World Journal of Gastroenterology*.

[21] Veréb Z., Póliska S., Albert R. (2016). Role of human corneal stroma-derived mesenchymal-like stem cells in corneal immunity and wound healing. *Scientific Reports*.

[22] Dominici M., le Blanc K., Mueller I. (2006). Minimal criteria for defining multipotent mesenchymal stromal cells. The International Society for Cellular Therapy position statement. *Cytotherapy*.

[23] Szabo A., Rajnavolgyi E. (2013). Collaboration of toll-like and RIG-I-like receptors in human dendritic cells: tRIGgering antiviral innate immune responses. *American Journal of Clinical and Experimental Immunology*.

[24] Kaundal U., Bagai U., Rakha A. (2018). Immunomodulatory plasticity of mesenchymal stem cells: a potential key to successful solid organ transplantation. *Journal of Translational Medicine*.

[25] Caplan A. I. (1991). Mesenchymal stem cells. *Journal of Orthopaedic Research*.

[26] Samsonraj R. M., Raghunath M., Nurcombe V., Hui J. H., van Wijnen A. J., Cool S. M. (2017). Concise review: multifaceted characterization of human mesenchymal stem cells for use in regenerative medicine. *Stem Cells Translational Medicine*.

[27] Nauta A. J., Fibbe W. E. (2007). Immunomodulatory properties of mesenchymal stromal cells. *Blood*.

[28] Covas D. T., Panepucci R. A., Fontes A. M. (2008). Multipotent mesenchymal stromal cells obtained from diverse human tissues share functional properties and gene-expression profile with CD146^+^ perivascular cells and fibroblasts. *Experimental Hematology*.

[29] Harkness L., Zaher W., Ditzel N., Isa A., Kassem M. (2016). CD146/MCAM defines functionality of human bone marrow stromal stem cell populations. *Stem Cell Research & Therapy*.

[30] Wangler S., Menzel U., Li Z. (2019). CD146/MCAM distinguishes stem cell subpopulations with distinct migration and regenerative potential in degenerative intervertebral discs. *Osteoarthritis and Cartilage*.

[31] Nombela-Arrieta C., Ritz J., Silberstein L. E. (2011). The elusive nature and function of mesenchymal stem cells. *Nature Reviews Molecular Cell Biology*.

[32] Kim K. C., Lee J. C., Lee H., Cho M.-S., Choi S. J., Hong Y. M. (2016). Changes in caspase-3, B cell leukemia/lymphoma-2, interleukin-6, tumor necrosis factor-alpha and vascular endothelial growth factor gene expression after human umbilical cord blood derived mesenchymal stem cells transfusion in pulmonary hypertension rat models. *Korean Circulation Journal*.

[33] Tottey S., Corselli M., Jeffries E. M., Londono R., Peault B., Badylak S. F. (2011). Extracellular matrix degradation products and low-oxygen conditions enhance the regenerative potential of perivascular stem cells. *Tissue Engineering Part A*.

[34] Beck B., Driessens G., Goossens S. (2011). A vascular niche and a VEGF-Nrp1 loop regulate the initiation and stemness of skin tumours. *Nature*.

[35] Najar M., Krayem M., Meuleman N., Bron D., Lagneaux L. (2017). Mesenchymal stromal cells and toll-like receptor priming: a critical review. *Immune Network*.

[36] Waterman R. S., Tomchuck S. L., Henkle S. L., Betancourt A. M. (2010). A new mesenchymal stem cell (MSC) paradigm: polarization into a pro-inflammatory MSC1 or an immunosuppressive MSC2 phenotype. *PLoS One*.

[37] Kyurkchiev D., Bochev I., Ivanova-Todorova E. (2014). Secretion of immunoregulatory cytokines by mesenchymal stem cells. *World Journal of Stem Cells*.

[38] Jiang M., Gao T., Liu Y. (2019). CD14 dictates differential activation of mesenchymal stromal cells through AKT, NF-*κ*B and P38 signals. *Bioscience Reports*.

[39] Kavanagh D. P. J., Robinson J., Kalia N. (2014). Mesenchymal stem cell priming: fine-tuning adhesion and function. *Stem Cell Reviews and Reports*.

[40] Wang M., Yuan Q., Xie L. (2018). Mesenchymal stem cell-based immunomodulation: properties and clinical application. *Stem Cells International*.

