# Supplementary figures and images for: Vessel Wall-Derived Mesenchymal Stromal Cells Share Similar Differentiation Potential and Immunomodulatory Properties with Bone Marrow-Derived Stromal Cells
Source: Stem Cells Int. 2020 Oct 21;2020:8847038. doi: 10.1155/2020/8847038 (PMC7596426; doi:10.1155/2020/8847038)

Supplementary figure 1.

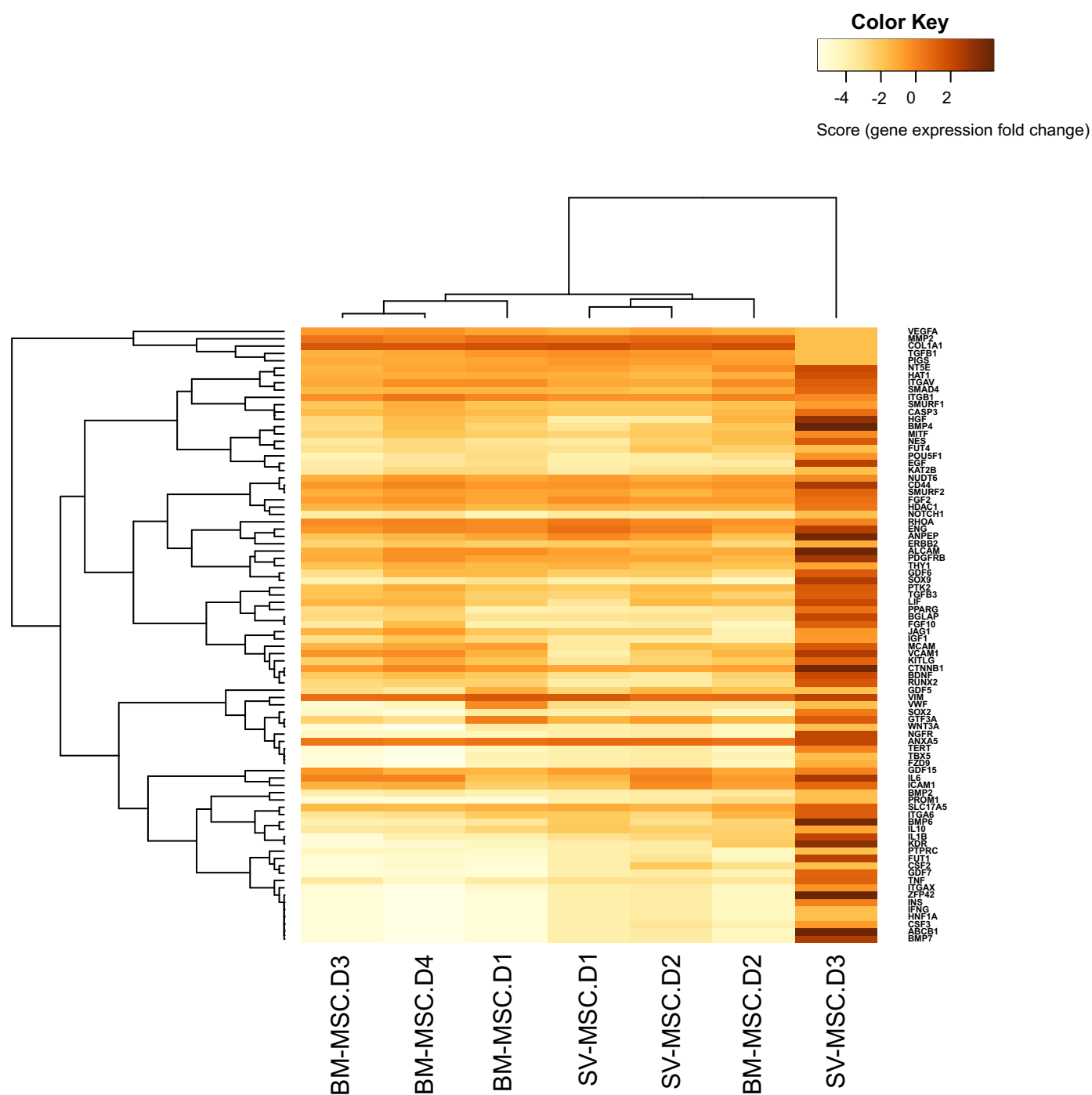

Supplement: Supplementary 1 — Figure S1: Heat map of differently expressed genes related to MSC and endothel cell biology. [file 8847038.f1.pdf]

Supplementary figure 2.

A

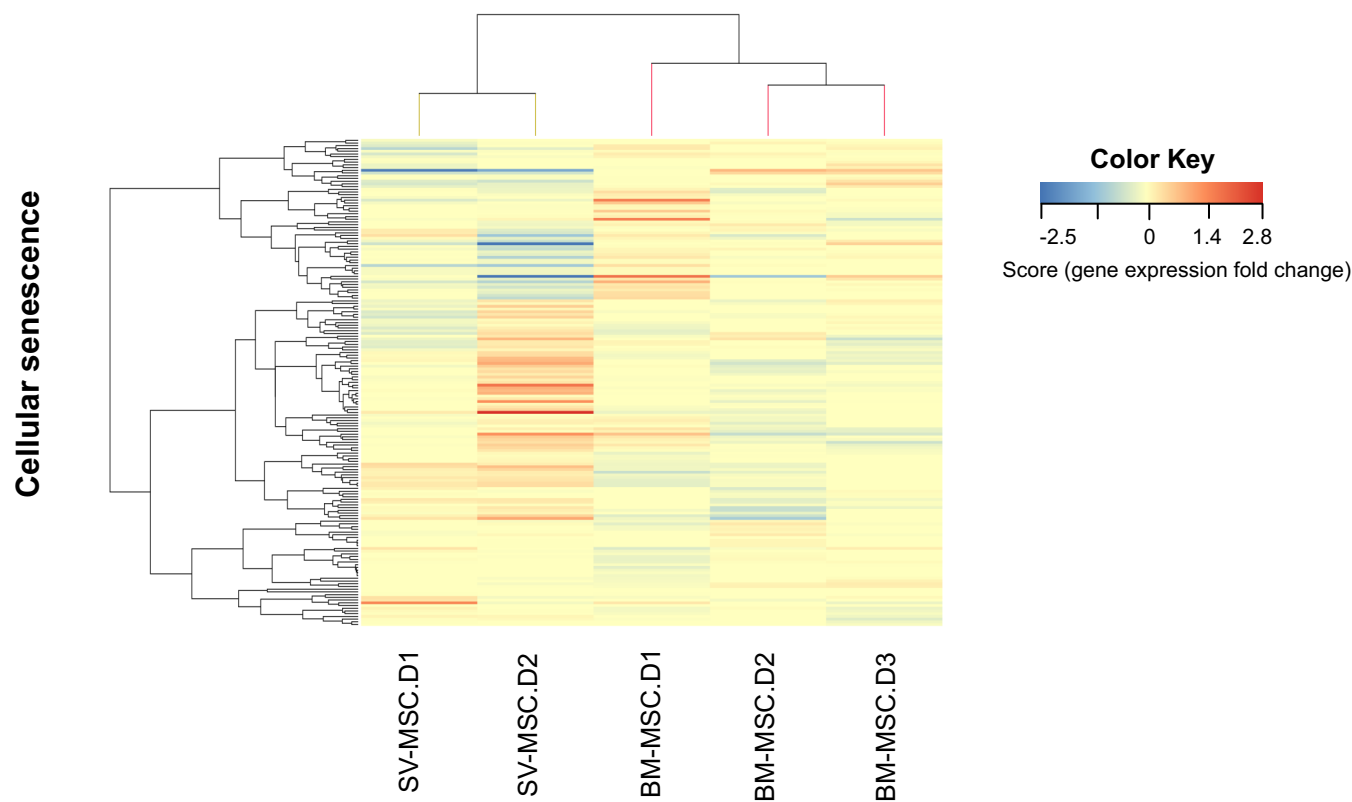

B

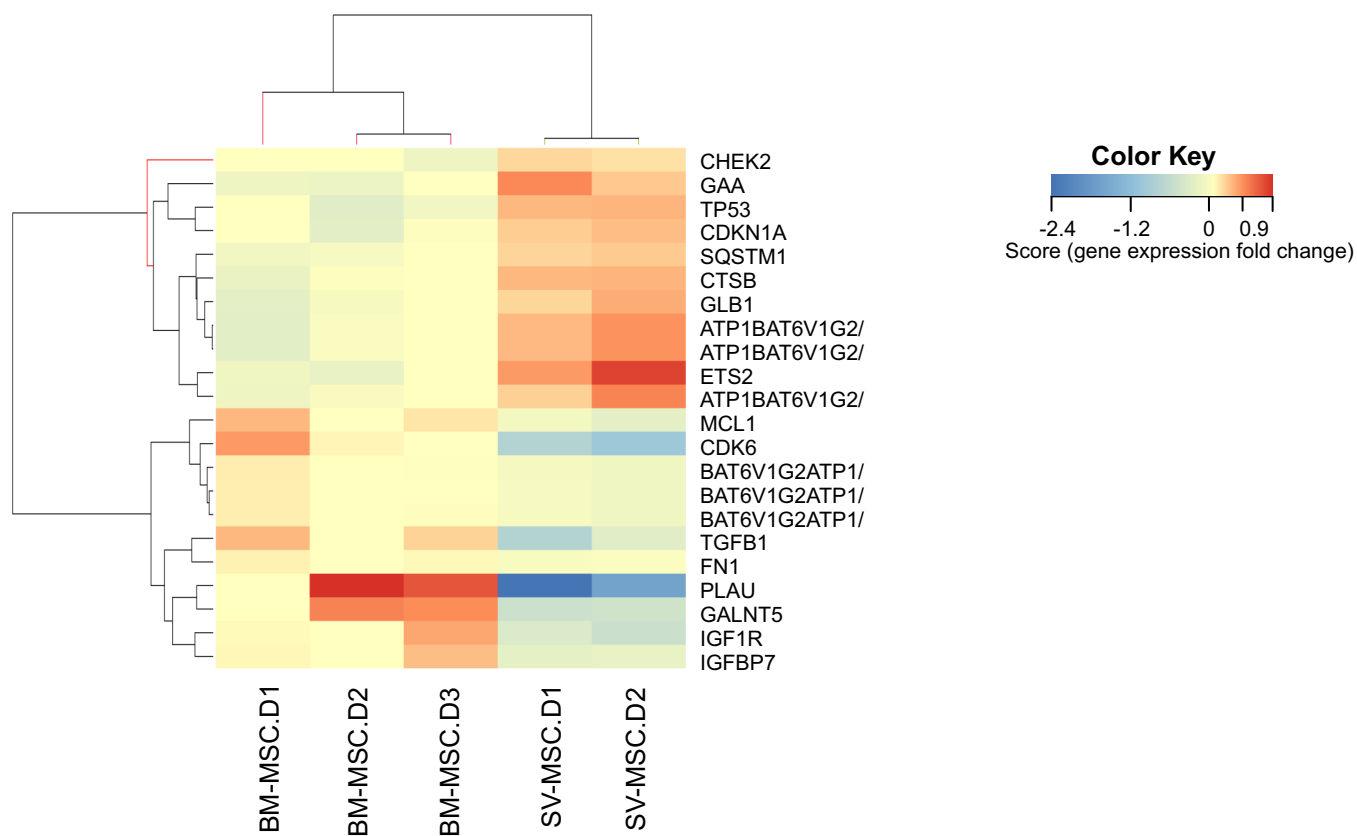

Supplement: Supplementary 2 — Figure S2: Expression of genes associated with cellular senescence [file 8847038.f2.pdf]

Supplementary figure 3.

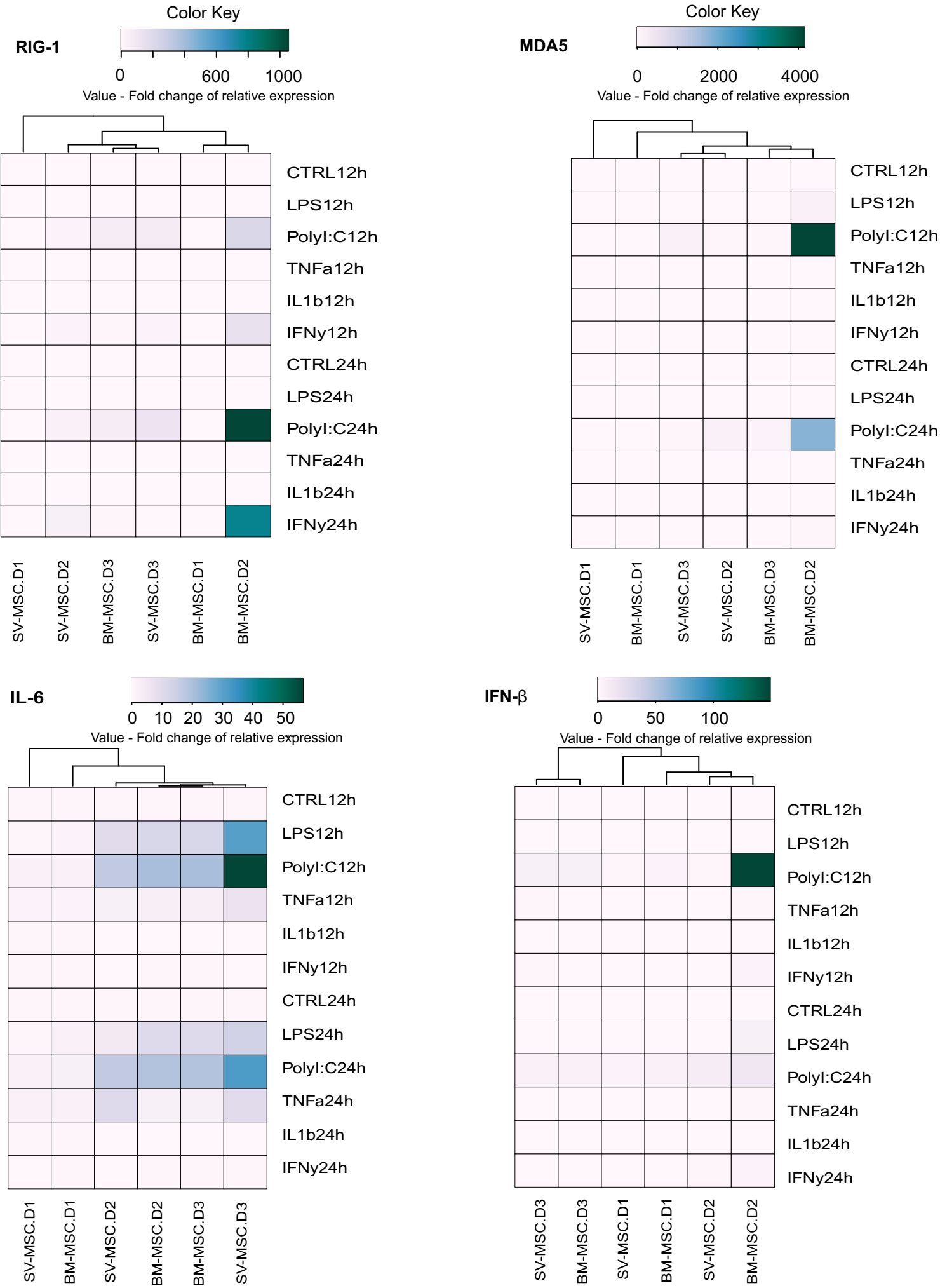

Supplementary figure 3.

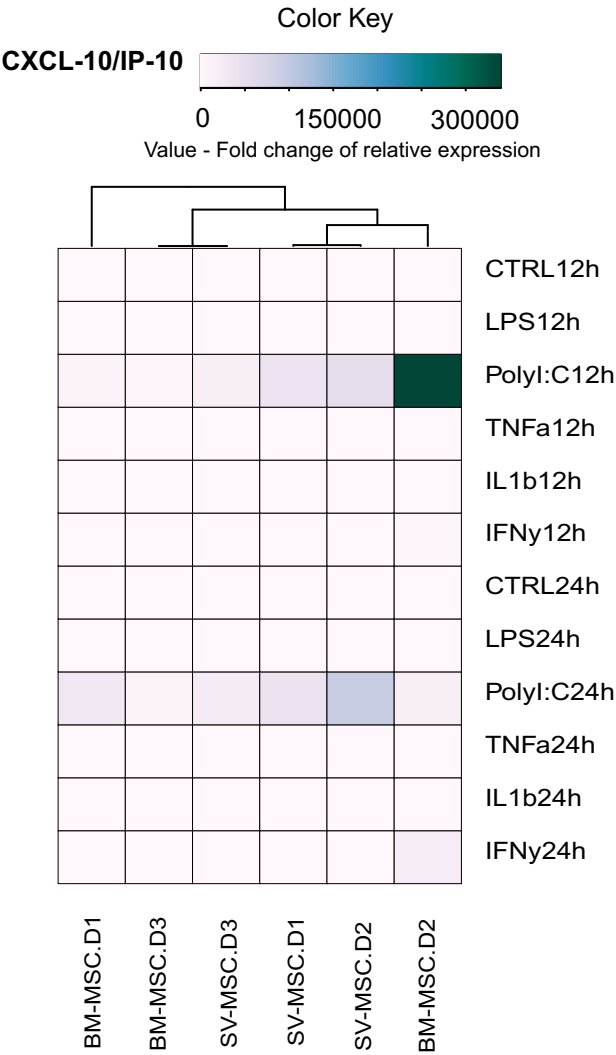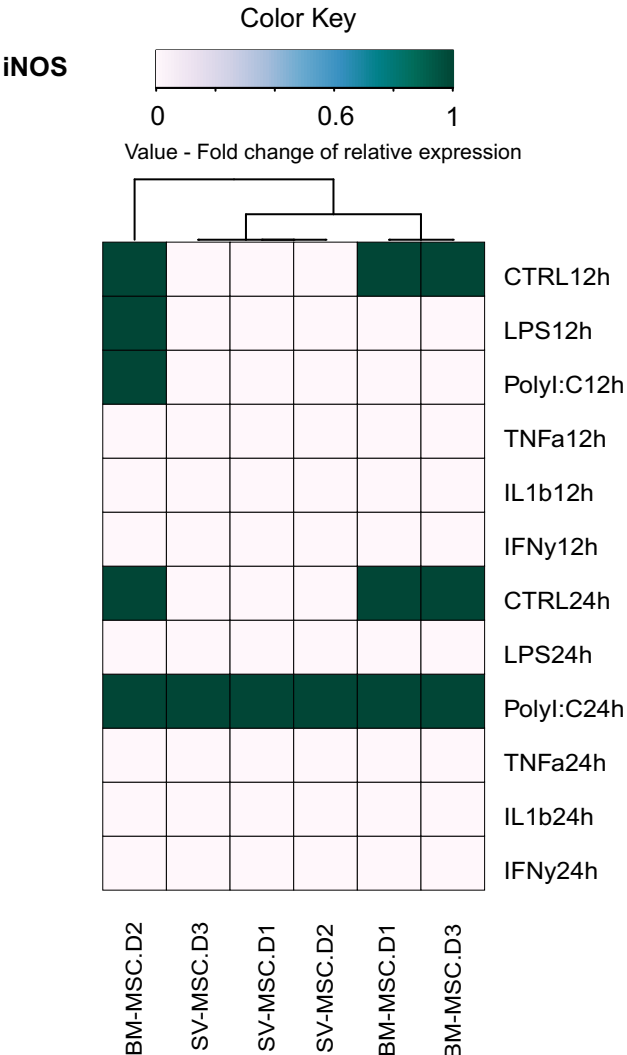

Supplement: Supplementary 3 — Figure S3: Heat maps of differently expressed genes related to TRL ligand- and proinflammatory cytokine-triggered activation. [file 8847038.f3.pdf]
