# Supplementary material for: Vessel Wall-Derived Mesenchymal Stromal Cells Share Similar Differentiation Potential and Immunomodulatory Properties with Bone Marrow-Derived Stromal Cells
Source: Stem Cells Int. 2020 Oct 21;2020:8847038. doi: 10.1155/2020/8847038 (PMC7596426; doi:10.1155/2020/8847038)
Supplement: Supplementary 4 — Supplementary Table 1: Significantly expressed genes associated with cellular senescence in SV-MSCs and BM-MSCs. [file 8847038.f4.docx]

**Supplementary information**

**Materials and methods**

Mesenchymal stromal cell-related gene expression profile was studied using the Human Mesenchymal Stem Cell PCR Array (SABiosciences). Total RNA was isolated by Trizol reagent, and then purified on RNeasy Mini kits (Qiagen) after DNase I digestion. RT2 First Strand kit was used to perform cDNA synthesis. Real-time PCR measurement was performed on the ABI Prism 7900 platform. Data were analyzed and statistical significance was calculated by SABiosciences on-line software.

**Supplementary Figure 1. Heatmap of differently expressed genes related to MSC biology.**

The clustering showed that cells isolated from the saphenous vein are MSCs, according the selected 84 transcripts. The mesenchymal stromal cell-related gene expression patterns were compared between SV-MSCs and BM-MSCs using Human Mesenchymal Stem Cell RT² Profiler™ PCR Array. The array includes 84 selected genes, which have previously been shown to play role in pluripotency of mesenchymal stromal cells.

**Supplementary Figure 2.** **Expression of genes associated with cellular senescence.**

A) The heatmap shows the expression of 160 genes that play a key role in cellular senescence. B) Heatmap of significant differences in the expression of senescence genes in BM- and SV-MSCs. The fold change values of gene expression at 2/3 of the genes do not even reach the threshold of 1.5 value.

**Supplementary Figure 3. Heatmaps of differently expressed genes related to TRL ligand- and pro-inflammatory cytokine-triggered activation.**

The heatmaps represent the fold change of the expression of selected genes that play a role in MSC activation. The hierarchical clustering analysis showed that there were no significant differences between SV-MSCs and BM-MSCs in the expression of any of the selected genes.

**Supplementary Table 1. Significantly expressed genes associated with cellular senescence in SV-MSCs and BM-MSCs.**

| **Transcripts Cluster Id** | **p ([SV-MSC] Vs [BM-MSC])** | **Regulation ([SV-MSC] Vs [BM-MSC])** | **FC ([SV-MSC] Vs [BM-MSC])** | **Gene symbol** | **Gene description** |
| --- | --- | --- | --- | --- | --- |
| 7919751 | 0.046034392 | down | -1,2449377 | MCL1 | myeloid cell leukemia sequence 1 (BCL2-related) |
| 7928429 | 0.003734809 | down | -5,8299394 | PLAU | plasminogen activator, urokinase |
| 7986383 | 0.015980612 | down | -1,51225 | IGF1R | insulin-like growth factor 1 receptor |
| 8010354 | 0.016304944 | up | 1,3709407 | GAA | glucosidase, alpha; acid |
| 8012257 | 0.017758789 | up | 1,3505553 | TP53 | tumor protein p53 |
| 8037005 | 0.022905849 | down | -1,6351124 | TGFB1 | transforming growth factor, beta 1 |
| 8045835 | 0.011269497 | down | -1,785591 | GALNT5 | UDP-N-acetyl-alpha-D-galactosamine:polypeptide N-acetylgalactosaminyltransferase 5 (GalNAc-T5) |
| 8058765 | 0.046284672 | down | -1,0501237 | FN1 | fibronectin 1 |
| 8068593 | 0.011230166 | up | 1,6429609 | ETS2 | v-ets erythroblastosis virus E26 oncogene homolog 2 (avian) |
| 8075164 | 0.049252987 | up | 1,1397212 | CHEK2 | CHK2 checkpoint homolog (S. pombe) |
| 8086028 | 0.038522486 | up | 1,2825501 | GLB1 | galactosidase, beta 1 |
| 8100541 | 0.024330916 | down | -1,2643099 | IGFBP7 | insulin-like growth factor binding protein 7 |
| 8110569 | 0.009813792 | up | 1,1974499 | SQSTM1 | sequestosome 1 |
| 8119088 | 0.04517005 | up | 1,2558943 | CDKN1A | cyclin-dependent kinase inhibitor 1A (p21, Cip1) |
| 8124926 | 0.014867996 | down | -1,1104634 | BAT1\|ATP6V1G2 | HLA-B associated transcript 1 \| ATPase, H+ transporting, lysosomal 13kDa, V1 subunit G2 |
| 8124942 | 0.020744938 | up | 1,3768737 | ATP6V1G2\|BAT1 | ATPase, H+ transporting, lysosomal 13kDa, V1 subunit G2 \| HLA-B associated transcript 1 |
| 8140955 | 0.003490548 | down | -2,101124 | CDK6 | cyclin-dependent kinase 6 |
| 8149330 | 0.015357438 | up | 1,2839022 | CTSB | cathepsin B |
| 8178476 | 0.019531826 | down | -1,1036656 | BAT1\|ATP6V1G2 | HLA-B associated transcript 1 \| ATPase, H+ transporting, lysosomal 13kDa, V1 subunit G2 |
| 8178508 | 0.026176374 | up | 1,3250538 | ATP6V1G2\|BAT1 | ATPase, H+ transporting, lysosomal 13kDa, V1 subunit G2 \| HLA-B associated transcript 1 |
| 8179750 | 0.02161786 | down | -1,1031046 | BAT1\|ATP6V1G2 | HLA-B associated transcript 1 \| ATPase, H+ transporting, lysosomal 13kDa, V1 subunit G2 |
| 8179762 | 0.020744938 | up | 1,3768737 | ATP6V1G2\|BAT1 | ATPase, H+ transporting, lysosomal 13kDa, V1 subunit G2 \| HLA-B associated transcript 1 |
